# Comparison of the effect of ginger and aloe vera mouthwashes on xerostomia in patients with type 2 diabetes: A clinical trial, triple-blind

**DOI:** 10.4317/medoral.23998

**Published:** 2021-06-20

**Authors:** Forouzande Badooei, Elham Imani, Saeid Hosseini-Teshnizi, Mania Banar, Mohammad-Reza Memarzade

**Affiliations:** 1 Master of nursing student, MSc student in medical-surgical nursing, Student Research Committee, Hormozgan University of Medical Sciences, Bandar Abbas, Iran; 2 Assistant Professor, Department of Nursing, Faculty of Nursing and Midwifery, Hormozgan University of Medical Sciences, Bandar Abbas, Iran; 3 Assistant Professor, Department of Biostatistics, Faculty of Nursing and Midwifery, Hormozgan University of Medical Sciences, Bandar Abbas, Iran; 4 Assistant Professor, Faculty of Medicine, Hormozgan University of Medical Sciences, Bandar Abbas, Iran; 5 Ph.D. in Chemistry, Barij Medicinal Plants Research Center, Isfahan, Iran

## Abstract

**Background:**

Ginger and aloe vera are two medicinal herbs mostly used to produce mouthwash. This study aimed to compare the effects of ginger and aloe vera mouthwashes on the xerostomia in patients referred to Bandar Abbas diabetes clinic (Iran).

**Material and Methods:**

In this triple-blind clinical trial, three groups (n=35 patients in each group) were studied. One group was given aloe vera mouthwash, the other group used ginger mouthwash, and the control group was only given normal saline. All the mouthwashes were used 20 ccs three times daily for 14 consecutive days. The symptoms and severity of xerostomia were measured before and after the intervention by the questionnaire proposed by Fox *et al*. as well as VAS scale, respectively. Statistical analysis, including the Shapiro-Wilk test used to measure the normality of variables, and Chi-square and McNemar used to compare the scores questionnaire proposed by Fox *et al*., were also applied. Scores of VAS were analyzed using a one-way ANOVA test. *P*<0.05 was considered as statistically significant in all these tests.

**Results:**

The mean age of the participants included in the normal saline group was 58.13±14.75 years old, 54.14±9.35 years old in the ginger group, and 53.37±11.57 years old in the aloe vera group. The difference between the scores of xerostomia before and after performing the intervention (The amount of reduction in xerostomia) in the ginger group was 6.12±2.004 cm, in the aloe vera group it was 4.08±2.09 cm, and in the normal saline group it was 2.45±2.09 cm. Finally, the ginger and aloe vera mouthwashes significantly reduced all symptoms and severity related to xerostomia, respectively(p<0.001).

**Conclusions:**

The use of mouthwash is an effective measure to reduce xerostomia under chronic conditions. Aloe vera, ginger, and normal saline are known as effective mouthwashes on controlling this complication. According to the results of this study, ginger and aloe vera mouthwashes could significantly decrease xerostomia and promote oral health in patients with diabetes.

** Key words:**Aloe vera, diabetes mellitus, dry mouth, ginger, mouthwash, xerostomia.

## Introduction

Diabetes mellitus is a chronic metabolic disorder characterized by elevated blood sugar as well as the impaired carbohydrate metabolism, fat, and protein.

The complications of diabetes significantly affect both the quality of life and longevity of the affected population, and also increase the related health care costs (1). Prolonged hyperglycemia causes some widespread systemic complications, including cardiovascular diseases, neuropathy and nephropathy, and oral complications. In this regard, oral complications of diabetes are xerostomia, mucous membrane irritation, tooth decay, periodontal diseases, delayed healing of oral ulcers (2), oral candidiasis, taste disturbance, geographical and cleft palate, neurological disorders, and benign Parotid hypertrophy (1). Correspondingly, Xerostomia is recognized as one of the main complications of diabetes. In one study, the prevalence rate of xerostomia in diabetic patients was estimated as 76.4% (3), which was 80% in another study (1).

Xerostomia is the subjective complaint on abnormal dryness of the oral cavity (2-4). In patients with diabetes mellitus, this feeling of dryness can occur due to a significant reduction, a lack of saliva production (3,5) or a change in saliva quality (3).

Furthermore, Xerostomia can lead to some complications, including infections and mouth ulcers, tooth decay, difficulty in speaking, difficulties in chewing and swallowing food, atrophic changes in the oral mucosa (3), respiratory problems and respiratory tract infections, and bacterial accumulation.

Bacterial accumulation in the oral cavity was found to be associated with some local and systemic complications such as stomatitis, periodontitis, sepsis, arthritis, and endocarditis (6). These effects could gradually lead to a severe decline in the quality of life of the patient at last (2,4,7,8).

A systematic review studied the pharmacological and non-pharmacological treatments of xerostomia.

In this study, xerostomia management interventions, including traditional medicine, pilocarpine, saliva substitutes, and chewing gum were investigated. The researchers concluded that these interventions have different levels of effectiveness on the management and prevention of dry mouth. However, in general, it seems that the existing interventions cannot provide any comprehensive, efficient, and long-term management in this regard (5).

These issues have led patients and health care providers to pay more attention to alternative and complementary medicines (5). One of the treatment alternatives for xerostomia in complementary medicine is the use of herbs (6). Ginger, as a medicinal plant with the scientific name of Zingiber officinale Rosco, belongs to the Zingiberaceae family. It is one of the most widely used spices worldwide. Ginger contains volatile oils, and its most important biological constituents are gingerols and shogaols (9). Moreover, ginger has some positive effects under various conditions, including nausea and vomiting, metabolic syndrome, pain relief (10), weight loss (9), anti-inflammatory, and antioxidant properties (11), and it can also be used in the treatment of many oral disorders (12). The presence of ginger in the oral cavity increases salivation and stimulates the salivary glands to produce more saliva (13).

Aloe vera is another medicinal plant with more than 75 active ingredients in its inner gel. About 99% of the gel in aloe vera leaves is water, so it has a strong moisturizing effect. Mucopolysaccharides in the gel help in binding the moisture to the skin and mucosa of the mouth. The studies conducted on the effect of ginger on xerostomia are limited, so further investigations are needed (4,14), Additionally, there is no study on the comparative effects of aloe vera and ginger on xerostomia so far. Besides, caring patients and maintaining their oral hygiene are the responsibilities of the nurse who is primarily responsible for helping a patient to maintain oral hygiene. As well, the prevalence of diabetes among the population is high, as about 2300 patients with diabetes currently go to Bandar Abbas Diabetes Clinic, and the percentage of oral complications caused by the disease, especially xerostomia, is also high. Therefore, in this study, we decided to use the potential of complementary medicine, in order to identify therapeutic or control methods of xerostomia. This research aimed to determine the effects of ginger and aloe vera mouthwashes on xerostomia in the patients with diabetes.

## Material and Methods

The present study was performed on 105 patients referred to the diabetes clinic in Bandar Abbas (Iran). In addition, this was a randomized, controlled, and triple-blind clinical trial. The inclusion criteria were patients with diabetes mellitus with xerostomia, non-allergic to ginger or aloe vera, consent to participate in the study, having no oral ulcers or infections, having no excessive physical activity, using no king of mouthwash or artificial saliva, and mental and physical ability to use the mouthwash. As well, unwillingness to continue the study, death, and migration were the exclusion criteria.

After approving the proposal by the Ethics Committee of Hormozgan University of Medical Sciences (IR.HUMS.REC.1397.299), the researcher identified 105 qualified subjects (n=35 in each group) for this study. The sampling method used in this study was availability sampling. The groups were assigned using a random number Table, and then we placed the participants in three groups receiving mouthwashes A, B, or C based on the randomized list. The blinding method was used for all three types of mouthwashes (ginger 25%, aloe vera 50%, and normal saline for washing) that were prepared by Barij Esans Company in the same color and packaging with different codes of A, B, and C.

The researcher, patients, and the statistical analyst were all unaware of which code belonged to which mouthwash and just one non-beneficiary person was aware of the contents of the bottles who revealed them after completing all the statistical analyses. Before performing the intervention, the patients completed a demographic information questionnaire proposed by Fox *et al*. (to determine the presence of xerostomia symptoms) (15). Moreover, the criterion for determining xerostomia was considered as the subjective sensation of abnormal dryness of the oral cavity (According to the definition of xerostomia) that was diagnosed by the questionnaire proposed by Fox *et al*.

The severity of xerostomia was also measured before the intervention using a visual analog scale (VAS) (16). According to similar studies performed in this field, the patients in all three groups used a 20ml mouthwash three times a day (preferably after their meals), kept it for one minute, and then poured it out (17). After the intervention, the patients once again completed the questionnaire proposed by Fox *et al*. as well as the VAS. Correspondingly, the validated version of the questionnaire proposed by Fox *et al*. in Iran contains 10 questions related to xerostomia, in which the response options are answered by “yes” and “no”. Each patient who answers at least four questions positively is considered as having xerostomia (18). Notably, the visual analog scale is linear. Accordingly, this standard questionnaire result is ranged from 0(no xerostomia) to 100 mm (severe xerostomia), and the patients are signed on the line. The reliability and validity of the two above-mentioned instruments were evaluated and then validated in the present study.

To evaluate the efficacy of mouthwashes in the respective groups, we measured the difference between the xerostomia scores based on VAS before and after the intervention.

Quantitative variables were reported by descriptive statistics indices and qualitative observations were presented using frequency distribution and percentages. Normality of the variables was estimated by the Shapiro-Wilk test. Chi-square and McNemar were also used to compare the scores obtained from the questionnaire proposed by Fox *et al*. before and after the intervention. In addition, the scores of VAS were analyzed using a one-way ANOVA test. Chi-square and one-way ANOVA tests were also used to compare demographic variables. *P*<0.05 was considered as statistically significant in all the tests.

## Results

After decoding, it was cleared that code A was for normal saline mouthwash, code B was for aloe vera mouthwash, and code C was for ginger mouthwash. The demographic characteristics of the participants included in these three groups before the intervention are compared in Table 1. The results of Chi-square and one-way ANOVA showed that these characteristics were homogeneous in all three groups. Most of the participants in these three groups were women. As well, most of the participants had other systemic diseases besides diabetes.

According to the questionnaire proposed by Fox *et al*., all the participants in this study had xerostomia. There was no significant difference among the three groups in terms of the xerostomia severity and symptoms before the intervention (*p*>0.05).

**Table 1:**
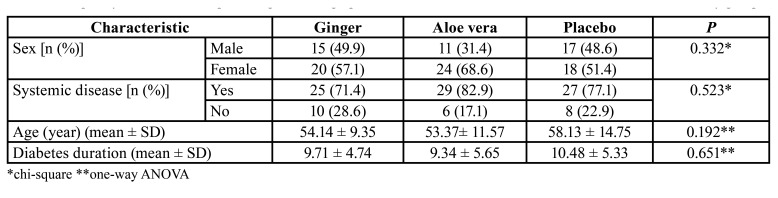
Frequency distribution and percentage of demographic characteristics of the individuals included in the three study groups.

In Table 2, we compared xerostomia symptoms after the intervention in the three groups. The Chi-square test showed that some xerostomia symptoms, including the need to drink water to swallow dry foods, sense of decreased salivation, experience of mouth dryness after waking up, feeling dry mouth during travel (*p*<0.001), and burning sensation in mouth (*p*<0.05) were significantly lower in the ginger and aloe vera groups compared to the control group. The results show that ginger and aloe vera mouthwashes could significantly reduce all the xerostomia symptoms (*p*<0.001).

One-way ANOVA showed that ginger, aloe vera, and normal saline in a descending order had the highest effects on decreasing xerostomia (Table 3). Additionally, we found no significant correlation among the variables of age, sex, and menopause commence and xerostomia (*p*>0.05).

**Table 2:**
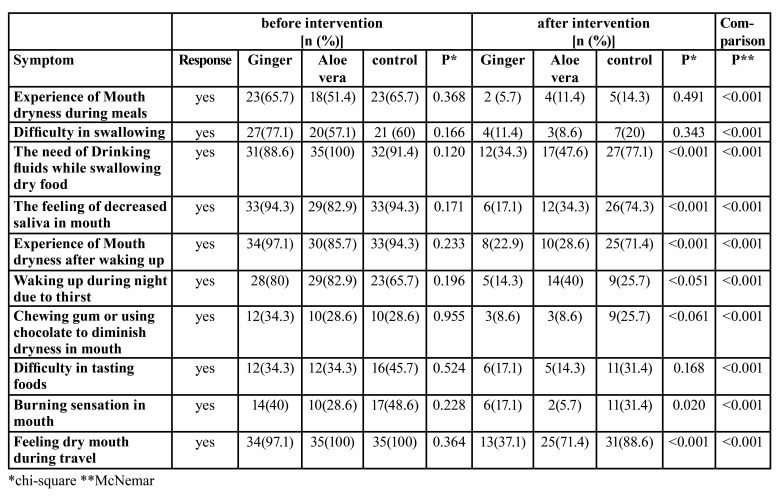
Comparison of xerostomia symptoms (the questionnaire proposed by Fox *et al*.) in the three groups before and after the intervention.

**Table 3:**
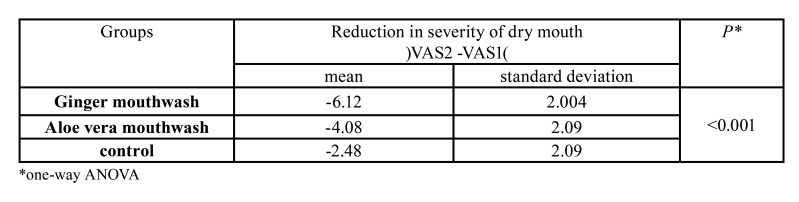
Comparison of the reduction of the xerostomia severity in the three groups.

## Discussion

As one of the oral manifestations of diabetes mellitus is xerostomia that creates many clinical and social problems for patients (1), this study aimed to determine the effects of ginger and aloe vera mouthwashes on xerostomia in patients with diabetes. According to the above-mentioned findings, the ginger mouthwash significantly reduced the symptoms and severity of xerostomia in the patients with diabetes mellitus in this study. Statistical analyses showed no significant difference among the three groups in terms of the severity and symptoms of xerostomia before the intervention. All the three groups were similar in age, sex, and duration of diabetes. This matter indicates that not only the three groups were completely identical before the study, but it also insists on the powers of both intervention and real random sampling.

The results of a study by Mardani showed that the mean salivary level in the intervention group significantly increased after ginger spray application (*p*<0.001), which consequently decreased the severity and symptoms of xerostomia (19). Accordingly, their results were consistent with those of the present study. In their research, the xerostomia of patients was due to diabetes, which was similar to the present study. The duration times of these two studies were almost the same. In the study by Nakayama, inhalation of both ginger and lemon oil improved salivary gland function and caused a significant increase in salivation in parotid glands before and after intervention (20). The results of this study also confirm the findings of the present study. However, we must not ignore the role of lemons in increasing saliva. The sample size and duration of intervention in the study by Nakayama *et al*. were similar to those of the present study.

There are very few studies conducted on the effects of aloe vera on xerostomia. Atashi *et al*.'s study confirm the results of the present study. Accordingly, they concluded that the aloe vera moisturizing gel significantly reduced xerostomia and improved oral health (6). In the study by Atashi, similar to the present study, aloe vera has been topically applied in the oral cavity. Their study recommended performing more studies with larger sample size and longer duration, which we did in the present study.

New methods of using complementary medicine to treat diseases and reduce their symptoms are currently under investigation. In the present study, the effectiveness of aloe vera mouthwash was found to be less than that of ginger. We suggest the use of aloe vera in future studies with non-mouthwash or mouthwash with higher concentration and percentage or in combination with artificial saliva.

The present study also had some limitations. As the first limitation, the patient used mouthwashes at home. To solve this problem, we reminded the patient or his/her family how to use the mouthwash every 2-3 days by phone; however, the researcher could not directly supervise the consumption. Another limitation was that the patients completed the questionnaire self-reportedly, so they may not have answered the questions correctly and accurately, which was beyond the researcher's control.

## Conclusions

The results show that ginger mouthwash can significantly reduce the xerostomia complication in patients with diabetes. Ginger has some special properties such as few complications, easy access, and cost-effectiveness. We suggest nurses to recommend this mouthwash to patients with dry mouth, in order to improve their oral health and subsequently improve their quality of life.
